# Substrate morphology modulates bacteriophage mobility and alters bactericidal potency

**DOI:** 10.1371/journal.pone.0356919

**Published:** 2026-09-16

**Authors:** Haoyu Wang, Oluyemi Olatunji Awolusi, Saravana Kumar Jaganathan, Jing Lyu, Chaozong Liu, Nick Tucker, Bukola Adenike Onarinde

**Affiliations:** 1 School of Engineering and Physical Sciences, University of Lincoln, Lincoln, United Kingdom; 2 School of Agri‐Food Technology and Manufacturing, University of Lincoln, Holbeach, United Kingdom; 3 Institute of Research and Development, Duy Tan University, Da Nang, Vietnam; 4 School of Engineering & Technology, Duy Tan University, Da Nang, Vietnam; 5 Biomedical Engineering Research Group, School of Engineering, University of Leicester, Leicester, United Kingdom; 6 Division of Surgery & Interventional Science, University College London, Royal National Orthopaedic Hospital, London, United Kingdom; University of Pennsylvania, UNITED STATES OF AMERICA

## Abstract

Bacteriophages (phages) are regaining attention as alternatives to antibiotics in the antimicrobial resistance crisis. Although successful cases are widely reported, the influence of physical, abiotic substrate morphology on phage performance remains largely unexamined, despite morphology being an intrinsic topographical feature present at many infection and decontamination sites, such as granular chronic wound beds or porous bone-implant interfaces. In this study, smooth, rough, and porous substrates were physically engineered to investigate the effects of abiotic substrate morphology on phage potency. Profiler and SEM analyses confirmed three distinct morphologies spanning several orders of magnitude in surface roughness. Phage K exhibited a clear hierarchy of bactericidal activity across these morphologies, with the highest activity on smooth substrates, reduced activity on rough substrates, and the lowest activity on porous architectures. Histological analysis and COMSOL simulations revealed that increasing morphological complexity elevates tortuosity and reduces phage accessibility and the probability of encountering pathogens. Together, these findings establish substrate morphology as a critical determinant of phage kinetics and bactericidal potency, relevant both to surface decontamination and, cautiously, to real infection sites such as wound beds and implant-tissue interfaces that inherently possess complex microarchitectures. Incorporating substrate morphology into the design of phage-based applications may improve the predictive accuracy of dosing, interval design, and clinical translation, and deepen understanding of the underlying pharmacology.

## 1. Introduction

An urgent call to address antimicrobial resistance (AMR) has been rising, driven primarily by the accelerating emergence of resistant pathogens, compounded by antibiotic discovery becoming increasingly slower [1–5]. Phages, as a promising solution, possess several advantages [6–12], such as a long record of clinical history, a rich genetic reservoir generated during co-evolution with bacteria, and high specificity for specific strains. Those advantages have attracted researchers to investigate phage-based approaches, both as decontamination tools and, more cautiously, as potential therapeutics against AMR [13].

Phage nanoscale structure plays a vital role in shaping its therapeutic properties. The size of the phage complex, consisting of a head with protein capsid enclosing genome, a proteinaceous tail and functional tail fibres for host recognition, ranges in dimension around 200 nm [14–16]. Phages are expected to behave as Brownian walkers in aqueous environments [17–19]. These investigations imply the local administration of phages is diffusion-limited and strongly dependent on the local substrate morphology. Nevertheless, the effects of abiotic substrate morphology on phage-bacteria interactions have not yet been investigated.

Tofu consists primarily of globulins, and it can form a three-dimensional dense, solid gel network under the action of coagulants [20,21]. Moreover, it can physically introduce distinct topological features, such as defined surface patterns and porous structures, via cutting, pressing, or freezing-thawing, without introducing additional chemical variations [22–24]. Tofu was therefore selected as a morphology-tunable substrate that reproduces the roughness and interconnected porosity features, allowing substrate morphology to be isolated as an experimental variable independent of host-tissue biochemistry. Bacteriophage K (Phage K) and its host *Staphylococcus aureus* (*S. aureus*) were selected. Phage K is a well-characterised phage of myophage morphology with an icosahedral ~50 nm head and a ~ 200 nm contractile tail [25,26]. Its host, *S. aureus*, is a Gram-positive pathogen. It shows a ~ 0.8–1.0 µm dimension and characteristic grapes-cluster morphology [27,28]. This pair represents an ideal model because *S. aureus* is frequently detected in clinical scenarios such as chronic wound beds [29–31], osteomyelitis [32–34], and the interface between implant and tissue [35,36], while Phage K shows highly selective recognition and rapid lysis of *S. aureus* [37–39].

This study reports for the first time that abiotic substrate morphology affects phage bactericidal potency. The *in vitro* model was engineered to exhibit distinct topological characteristics, including flat, rough and porous, followed by *S. aureus* colonisation and subsequent Phage K treatment. A potency hierarchy was observed, with smooth surfaces outperforming rough and porous. This study suggests incorporating substrate morphology into the framework of phage kinetics, thereby offering new perspectives for phage-based surface decontamination and other applications.

## 2. Materials and Methods

### 2.1. Materials

Tofu (Tofu King, Birmingham, UK) was selected as texture model material. *Staphylococcus aureus* and its lytic phage K were obtained from NCTC. *S. aureus* selective nutrient medium, Mannitol Salt Agar, was purchased from OXOID. Tris(hydroxymethyl)aminomethane (Tris) buffer, ethanol, glutaraldehyde (25% v/v, GLA), and hexamethyldisilazane (HMDS) were purchased from Sigma-Aldrich. Paraffin wax and xylene were obtained from Leica. 4′,6-diamidino-2-phenylindole (DAPI) and Remel™ Gram Stain Kit were purchased from Thermo Fisher Scientific (UK).

### 2.2. Phage K and *Staphylococcus aureus* preparation and characterisation

The lytic phage K (NCTC 7814) and its host *Staphylococcus aureus* (NCTC 9318) were employed in this study. Bacteria were cultivated and used in mid-logarithmic growth phase (OD600 0.2–0.4). For phage propagation, bacterial cultures were infected with phage K and incubated until visible lysis was achieved, as indicated by a decline in optical density. The lysate was subsequently clarified by centrifugation and filtration through a 0.22 µm membrane to remove cell debris. Phage stocks were adjusted to 1 × 10⁸ PFU/mL and titred using plaque assays. Both bacterial and phage cultures were used fresh; phage were titred by plaque assay before each experiment.

For transmission electron microscopy (TEM), phage suspensions (>10⁹ PFU/mL) were deposited as droplets and incubated with TEM grids for attachment, followed by staining with 2% uranyl acetate and three times washing. SEM was used to observe *S. aureus*, which was fixed and processed as described below.

### 2.3. Engineered substrate morphology

Substrate morphology was engineered on fresh tofu blocks to obtain smooth, rough, and porous morphologies. Smooth substrates were prepared by trimming tofu with a sterile scalpel. Rough textures were produced by pressing the cut surfaces against coarse fabric. Porous structures were generated by freezing at −20 °C overnight and thawing at room temperature. Engineered substrates with distinct topographic features were freshly prepared and autoclaved prior to antimicrobial testing.

For SEM analysis [40], engineered substrate samples were fixed in 2.5% glutaraldehyde (v/v) for 2 h at room temperature, dehydrated through a graded ethanol series (30%, 50%, 70%, 90%, 100%, 15 min each), treated with HMDS, sputter-coated with gold, and imaged using a scanning electron microscope (JEOL Ltd., Tokyo, Japan) at an accelerating voltage of 5 kV.

Porosity was determined by the liquid displacement method using absolute ethanol as the infiltrating medium. Engineered tofu blocks were immersed in ethanol within a graduated cylinder, and volume displacement was recorded (n = 3).

Surface morphology was measured using a KLA-Tencor (CNTec Instruments Co., Ltd., Milpitas, CA, USA) surface profiler with a scan scale of 0.25 mm per division. Each measurement was performed over a total scan length of 5 mm. Surface roughness parameters (Ra, Rq, and Rz) were calculated from the acquired height profiles, with three independent measurements (N = 3) obtained for each sample.

### 2.4. Substrate morphology effects on phage-bacteria interactions

Engineered substrate cubes (2 × 2 × 2 cm^3^) with defined surface topography were inoculated with *S. aureus* suspensions at 5 × 10^3^ CFU and incubated at room temperature for 30 min to allow bacterial attachment. Phage K suspensions were then administered at a titre of 1 × 10⁸ PFU (MOI ≈ 2 × 10⁴) and incubated at room temperature for 15 min. In parallel, a phage-free control was performed. Bacterial reduction was measured by agitating the samples in SM buffer using a Stomacher for 2 min, and 100 µL aliquots were plated on *S. aureus*-selective agar in three biological replicates to quantify and confirm the identity of surviving bacteria.

To understand the bacteria distribution, tofu samples were fixed and dehydrated as described above, embedded in paraffin, and sectioned. Sections were stained with Gram stain kit and DAPI to visualise bacterial distribution within the tofu matrix. Imaging was performed using a fluorescence microscope (Zeiss, Axioscope 5, Germany). SEM was applied to observe the cross-sections of the substrate.

COMSOL Multiphysics (6.4 COMSOL, Stockholm, Sweden) was used for simulation, and the 2D axisymmetric framework was applied. Laminar Flow and Particle Tracing for Fluid Flow physics interfaces were coupled using the Fluid-Particle Interaction multiphysics coupling to simulate solution spreading and phage particle mobility. For the laminar-flow model, the boundary in contact with the substrate was set as the stick wall, while the air-liquid interface was set as an Open Boundary. Phage administration was represented by an inlet with a prescribed velocity of 1 cm s ⁻ ^1^. To simplify computation, 1000 particles were released with random positions at the administration inlet. Particle motion was analysed by drag, gravity, and Brownian force. The aqueous phase was assigned the density and dynamic viscosity of water at room temperature. Surface geometries for the rough and porous models were digitised from the profiler height-maps, so that the simulated topography matched the measured substrates. A triangular mesh was used, refined at substrate boundaries to resolve the surface features. Streamlines of the phage solution spreading were obtained under stationary studies, and the resulting flow field was then used in a time-dependent study to compute particle mobility. Representative particle mobility images were extracted to visualise retention and stagnant regions across morphologies.

### 2.5. Statistical analysis

All results are presented as mean ± standard deviation (SD) from at least three independent experiments (n = 3). Statistical analyses and plotting were performed using Origin software (Origin Pro 2024B, OriginLab, Northampton, MA, USA) and GraphPad (Boston, Massachusetts, USA) and BioRender. For quantitative characterisation data, such as pore size, surface roughness, and porosity, t-test was applied. For phage bactericidal evaluation, two-way ANOVA with Tukey’s post-hoc test was applied. Statistical significance was considered at p < 0.05, with *, **, and *** corresponding to p < 0.05, p < 0.01, and p < 0.001, respectively.

## 3. Results

### 3.1. Phage and host

**Fig 1:**
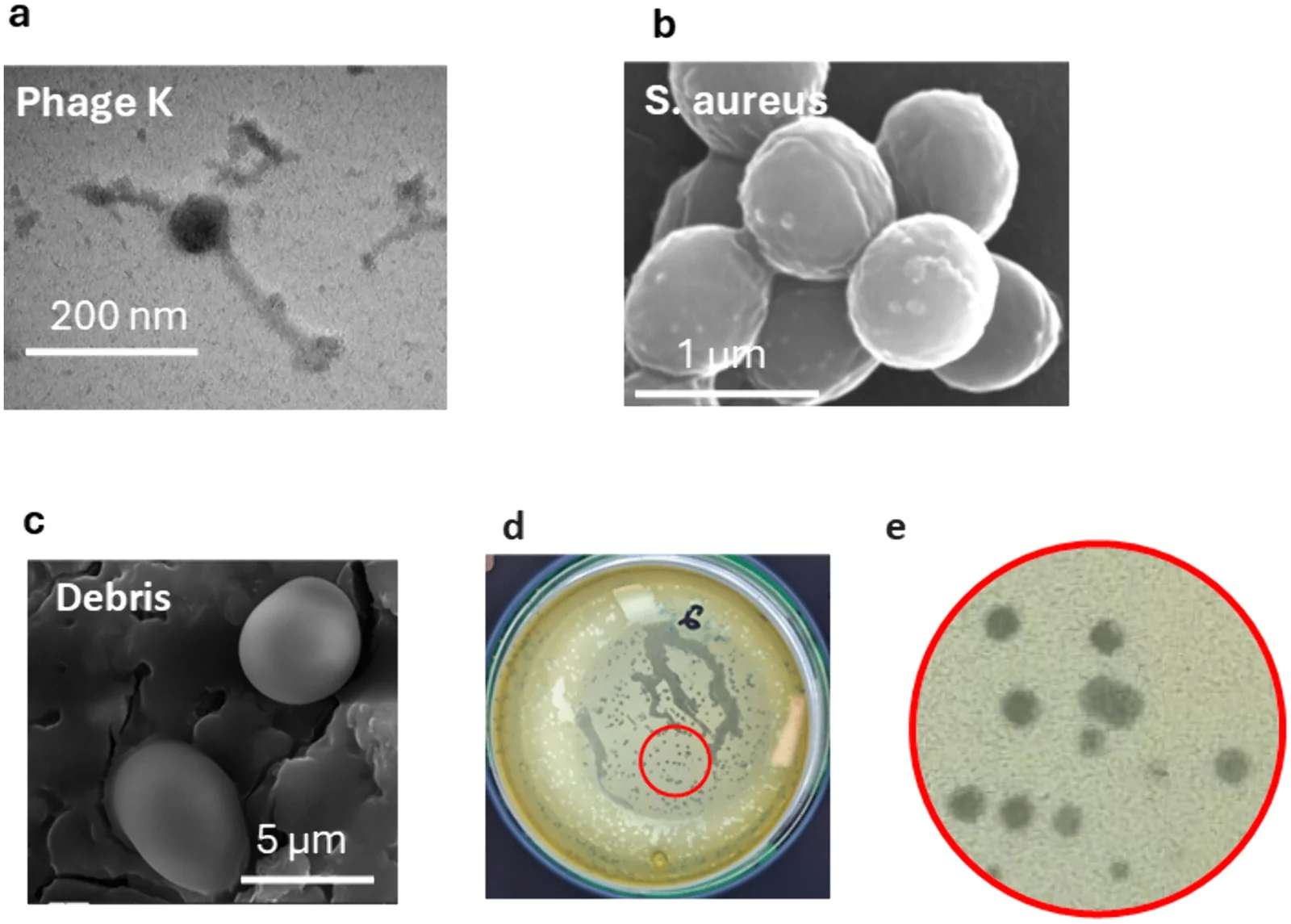
Characterisation of phage K and *S. aureus.* (a) Transmission electron micrograph of phage K. (b) Scanning electron micrograph of *S. aureus*. (c) Scanning electron micrograph of *S. aureus* following 30 min of Phage K treatment. (d) Double-layer agar plaque assay. (e) Higher-magnification view of the plaque boundary.

*S. aureus* and phage K were selected as the test model as shown in Fig 1. Phage K TEM image (Fig 1a) demonstrated typical myophage morphology, including a head around 50 nm and a contractile tail with around 250 nm length. *S. aureus* SEM images (Fig 1b) demonstrated typical grape-like clusters with 0.86 ± 0.06 µm size and rough surfaces with undefined nodes, suspected to be caused by dehydration and collapse of the membrane. Lysis was observed (Fig 1c) after 30 min phage treatment, with debris accumulation and swollen *S. aureus* observed, indicating bactericidal activity. This was also confirmed with the double-layer plaque test (Fig 1d), with a complete lysis zone producing a sharply demarcated, optically transparent region (Fig 1e). Hence, Phage K and its host, *S. aureus*, demonstrated effective interaction and bactericidal activity, and were selected as the model to research substrate texture effects on their interaction.

### 3.2. Engineered substrate morphology

**Fig 2:**
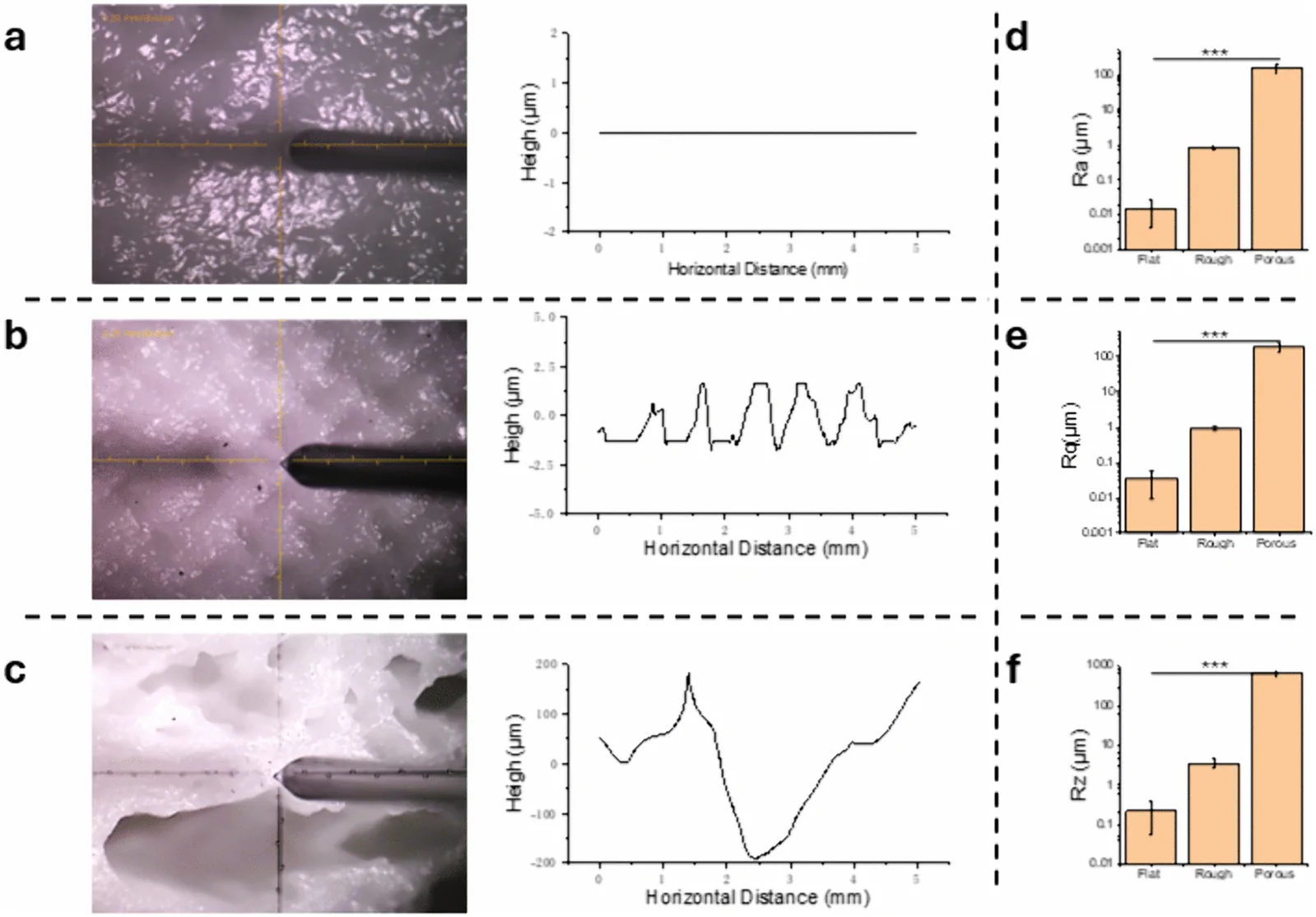
Surface texture engineering. Photo and corresponding line-scan height profiles of a) smooth, b) rough and c) porous surfaces. The roughness analyses of d) Ra, e) Rq, f) Rz. ‘***’ means p < 0.001 and N = 3.

Three distinct textures were made and characterised with a profiler for the following phage bactericidal potency test (Fig 2). The smooth substrate demonstrated a continuous flat surface with height fluctuation less than 1 µm (Fig 2a). While the rough substrate showed micro-scale regular undulations across the surface (Fig 2b). Its line-scan profile recorded wave-like height variation with around a 1 mm period and 2.5 µm height. Further, the interconnected porous substrates were crafted, with profiler imaging showing large, irregular cavities generated by the freeze-thaw process (Fig 2c). The Ra increased markedly from 0.01 ± 0.01 µm for smooth surfaces to 0.82 ± 0.10 µm for rough surfaces, and further to 158.88 ± 49.60 µm for porous substrates (Fig 2d). Similarly, the Rq (Fig 2e) demonstrated 0.03 ± 0.02 µm (smooth) to 0.96 ± 0.11 µm (rough), and reached 193.07 ± 59.57 µm in porous samples; the same trend was observed in Rz. Together, these data confirm the successful fabrication of three substrates of highly distinct morphology with order-of-magnitude differences in surface characteristics via roughness, and were used as a model for subsequent investigations into how substrate texture modulates phage potency.

### 3.3. Substrate morphology effects on phage bactericidal potency

**Fig 3:**
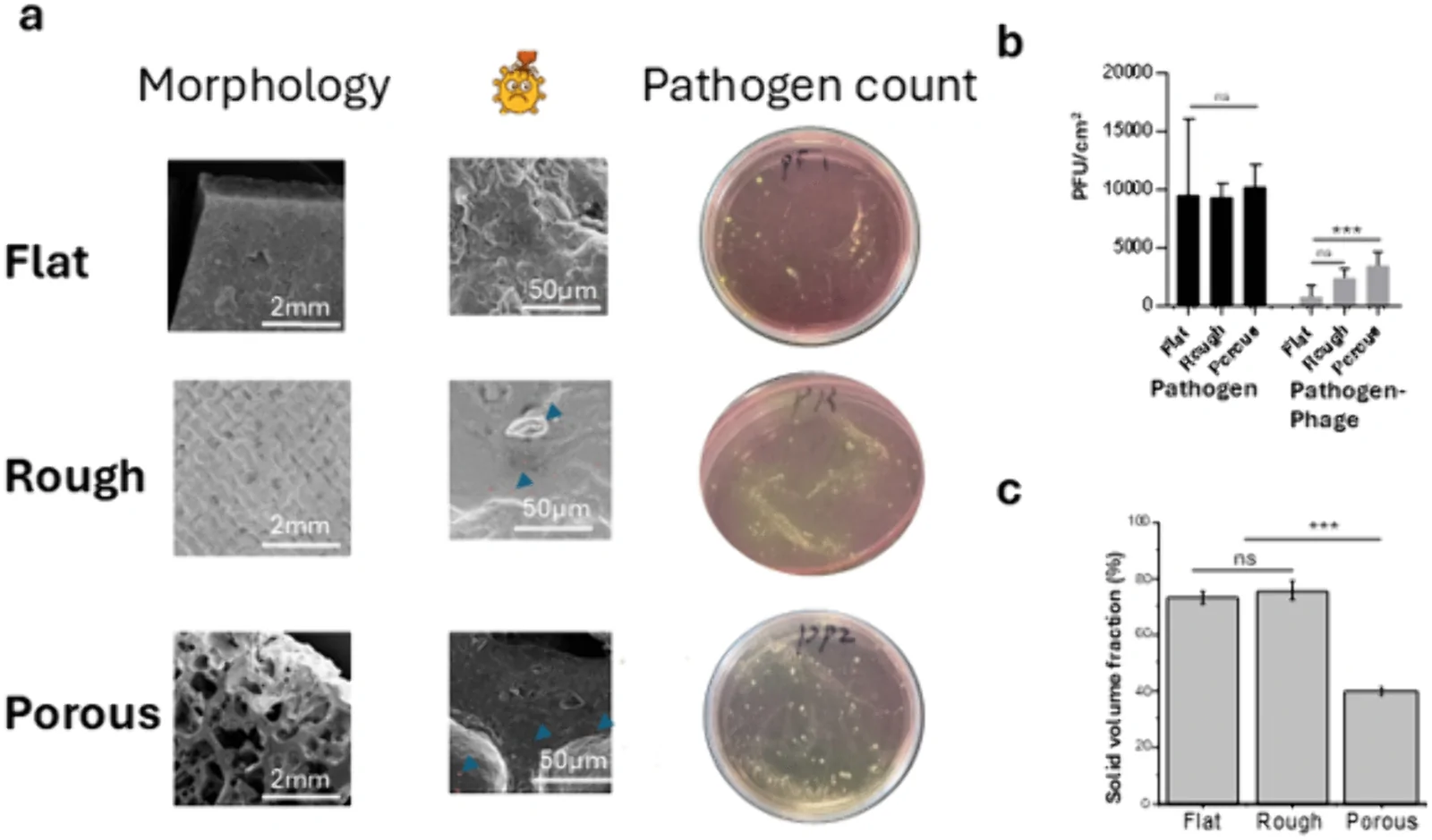
Morphology effects on pathogen clearance. a) SEM characterisation of flat, rough, and porous tofu textures (left), corresponding SEM images of *S. aureus* clearance on each texture rendered in pseudo-colour (pink) with blue arrows (middle), and representative outcomes of the phage-clearance assay on each texture type (right). b) Remaining pathogen counts (CFU/cm²), plated on *S. aureus*-selective agar (N = 3), shown for phage-free controls. c) Porosity measurements of the three textures. ‘***’ means p < 0.001; ‘ns’ means no significant difference. (N = 3).

Phage potency was investigated via phage administration on flat, rough, and porous engineered substrates as shown in Fig 3. The morphologies remained consistent with the profiler information, in which continuous smooth surfaces were observed on flat substrates, while periodic fluctuations were observed on rough substrates, and spongy structures were shown on porous substrates (Fig 3a). After the colonisation of *S. aureus* and the following administration of phage K, flat surfaces showed effective bactericidal activity with a cleared surface, and sporadic colonisation was observed on the selective agar plate for *S. aureus*. While on rough and porous substrates, extensive bacterial accumulation was observed on SEM images, which remained consistent with the corresponding *S. aureus* selective plate. The highest pathogen clearance efficiency was observed on the flat substrate with >99% bacteria killed, while on the rough and porous substrates 1525 ± 1025 and 2433 ± 573 CFU/cm^2^ were counted respectively (Fig 3b). In the phage-free control, bacterial recovery was 9617 ± 5282, 9333 ± 1009, and 10225 ± 1325 CFU/cm^2^ for flat, rough, and porous substrates respectively, with no significant difference between morphologies, implying that substrate morphology does not by itself alter bacterial recovery. These differences correlated with the intrinsic porosity and texture. Flat and rough substrates exhibited similar porosity levels at 28%, whereas porous substrates displayed a significantly lower solid volume fraction, around 40% porosity (Fig 3c).

### 3.4. Substrate morphology effects on bacterial colonisation

**Fig 4:**
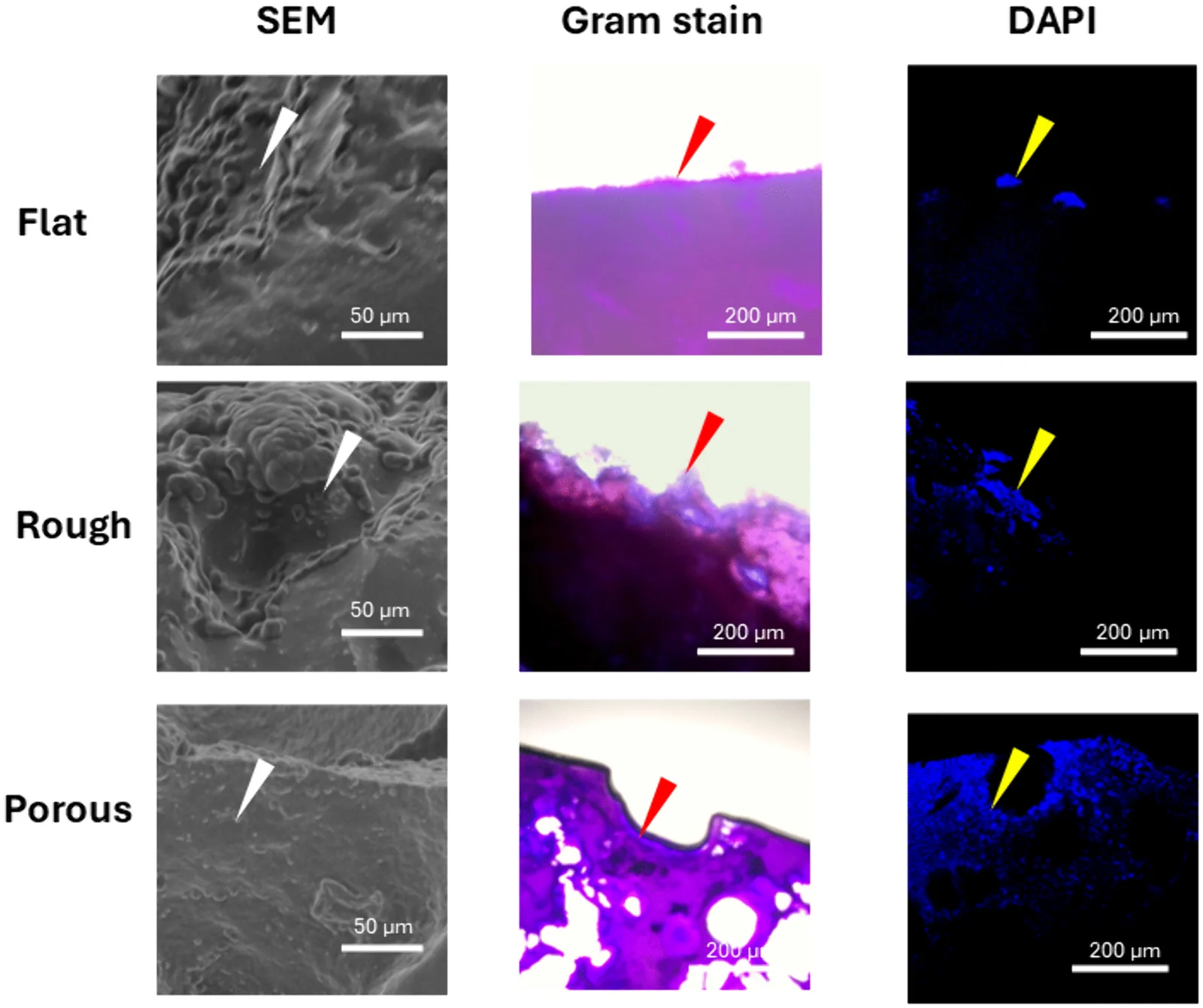
Texture-dependent spatial distribution of *S. aureus* on flat, rough and porous substrates, with SEM imaging, Gram and DAPI staining. Arrows indicate the locations of *S. aureus* accumulation.

The substrate morphology significantly changed the spatial distribution of colonising pathogens, as shown in Fig 4. On smooth substrates, *S. aureus* colonisation was limited to the surface, forming an accumulation but not a confluent layer. Correspondingly, Gram and DAPI staining images indicated only a superficial layer of bacteria. Although rough substrates also confined bacteria on the surface, the *S. aureus* clustered among those micro pits and irregular grooves. Both Gram and DAPI staining indicated the pocket-like colonisation of pathogen with complex spatial structure. In contrast, the most infiltrated colonisation was observed in porous substrates. The SEM image focused on an interconnected cavity wall, where *S. aureus* colonisation was widely distributed. Gram-stained sections displayed bacteria throughout the matrix porous network. While the bacteria used could not penetrate into the substrate, its microstructure affected bacterial spatial distribution, where phage accessibility may also be affected, given its nanoparticle mobility.

### 3.5. Substrate morphology effects on phage mobility

**Fig 5:**
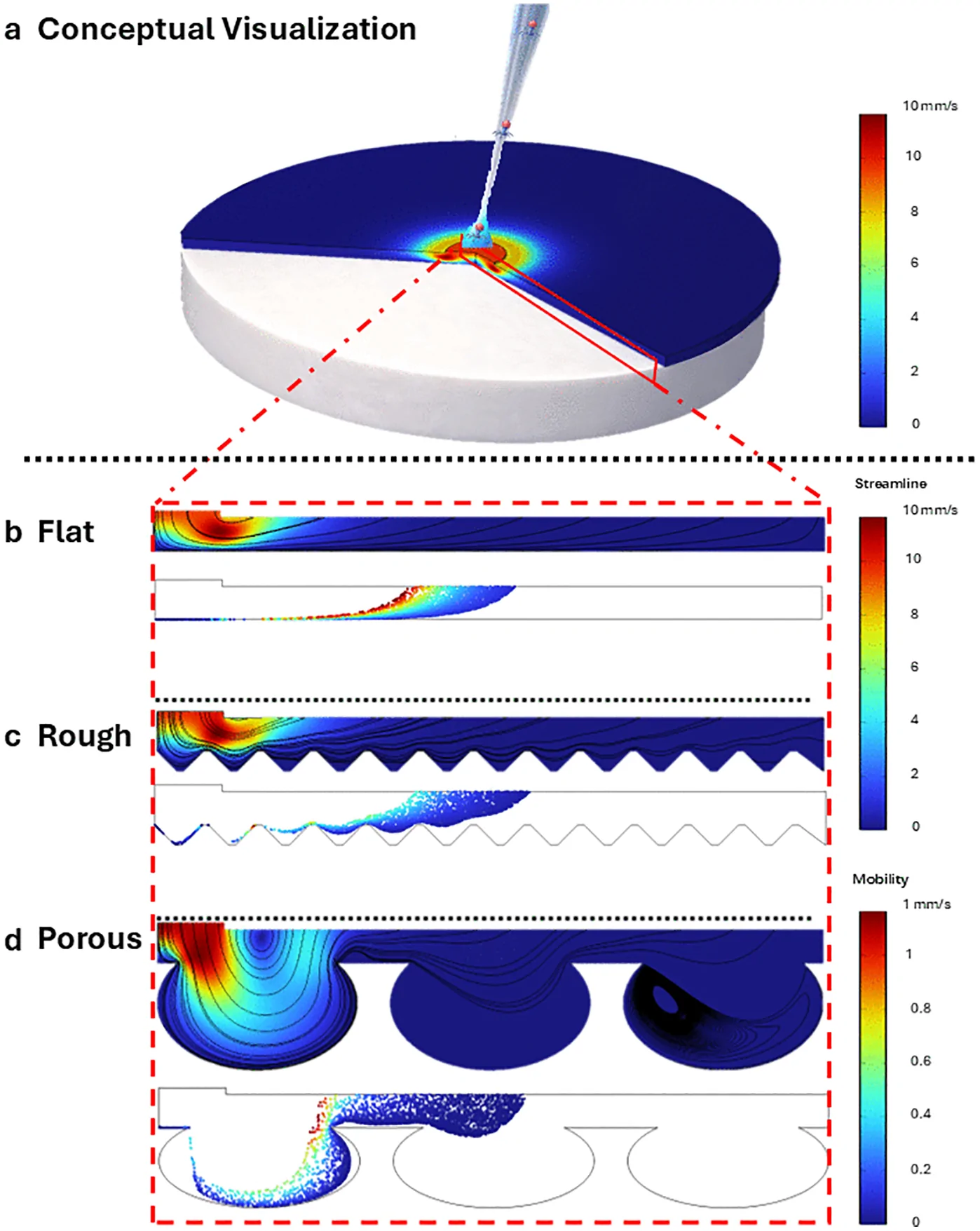
Simulation of morphology-dependent phage mobility. a) Schematic diagram of the model setup for simulation. b) Cross-sectional streamlines for flat, rough, and porous model, corresponding to phage mobility simulation.

Fig 5a shows the simulation setup, using the flat model as a representative example, applied to model phage solution spreading over the substrate surface to evaluate texture effects on phage mobility. Phages were set as nanoparticles, tracking the mobility in the aqueous phase, and were added at the inlet from a pipette.

For the flat model (Fig 5b), the streamline image shows smooth and uniform movement pathways of the phage solution, almost parallel to the surface. The simulation of phage particles demonstrated minimal geometric obstruction to phage motion, in which the phage particles exhibited lateral diffusion, covering the surface of the model and indicating a high encounter probability with bacteria colonised on the surface. While the condition of the rough model demonstrated partially confined mobility (Fig 5c). The streamlines exhibit local deviations and perturbations, with fluid transport along the surface irregularities. The simulated phage particles displayed hindered lateral diffusion at the far end, demonstrating transient dead zones without surface attachment, leading to reduced coverage efficiency and a lower probability of access to bacteria. A further restricted mobility was computed in the porous model (Fig 5d), in which the highly tortuous surface hindered the mobility of phage particles, giving rise to distinct retention zones near the site of phage administration and stagnant regions at the far end.

## 4. Discussion

This study identifies substrate morphology as an important factor influencing phage bactericidal performance. The findings suggest that phages, as nanoscale biological particles, are affected by the microstructural environment in which they are administered. This consideration is relevant to complex application sites, including granular wound beds, porous bone–implant interfaces, and structured food or device surfaces. Neglecting local substrate morphology may therefore lead to overestimation of phage accessibility and bactericidal efficacy.

The observed hierarchy of phage potency is attributed to substrate morphology altering phage mobility and, consequently, the probability of phage–pathogen encounters. Phages kill pathogens upon encounter, while texture affects both bacterial spatial distribution and phage mobility (Fig 4 and Fig 5). On flat surfaces, bacteria are distributed within a two-dimensional plane and phage particles diffuse freely across the planar interface without encountering physical barriers. Therefore, phage-pathogen adsorption occurs rapidly, killing >99% *S. aureus*. Rough substrates introduce micro pits, and bacteria colonise within wall-like niches that generate partial shielding effects. These pit structures reduce phage mobility, leading to a slowed but still effective phage potency. For the porous architecture, where *S. aureus* colonises across the full thickness, its interconnected pore network creates a three-dimensional microenvironment. Within these confined geometries, phages undergo significant retention due to the extended path, hence reduced encounter probability between phage and pathogen.

These findings extend the limited research on phage bactericidal potency. The effects of texture on bacterial behaviour during the infection process have been well investigated [41], with studies revealing that a non-smooth surface enhances bacterial colonisation by providing more anchoring sites, facilitates biofilm formation by reducing shear forces, and shields bacteria by creating an immune-evasive microenvironment. In contrast, the impacts of surface features on phages remain poorly understood [41–43]. Only a few studies have reported incidental observations between surface area and phage adsorption. For example, Vonasek et al. (2017) reported electrospun cellulose acetate with a high volume-to-area ratio non-specifically immobilized more phages, indicating that larger matrix surface area provides a higher phage retention rate [44]. Thus, while texture has long been recognised as an important factor of bacterial behaviour, its role in modulating phage mobility and further interactions between phages and bacteria remains largely unexplored.

Notably, disclosing the link between phage potency and substrate morphology has implications beyond decontamination. This work indicates that constraints of morphology at an administration site affect phage mobility, which warrants further evaluation and consideration of the morphology factor in phage applications more broadly. For example, in chronic diabetic foot ulcers (DFUs), the wound bed exhibits a granular, heterogeneous surface [45]. Similarly, in bone-implant interface infections, the trabecular bone zone at the interface presents a highly porous architecture, with pore sizes typically ranging from 50–500 µm and interconnectivity reaching 60–90% [46–48]. For both scenarios, the morphology effects on phage potency are currently overlooked. Hence, accounting for morphology constraints at the administration site is essential for further developing phage-based applications, including phage therapy where relevant.

This study also carries broader implications in food disinfection and device sanitation. For instance, medical devices such as interbody cages [49,50], 3D-printed bone scaffolds [50] or electrospun devices [51,52] have complex geometry which may affect the use of phage as a disinfection method. Likewise, food also presents topographical diversity [53], ranging from smooth salmon to highly irregular hierarchical broccoli, which will also challenge phage application in food decontamination.

Finally, this study has several limitations. First, COMSOL particle-tracing simulations were applied to explain the bactericidal differences, and the simulation was not a direct method to measure phage mobility; the proposed mechanism should therefore be regarded as a hypothesis. Second, the tofu substrate is a simplified model, employed here to isolate substrate morphology as an experimental variable. The present findings therefore suggest that morphology, as a determinant of phage mobility and potency, should be investigated using biologically relevant matrices, such as collagen-based hydrogels, *ex vivo* wound tissue, or biofilm-laden substrates. Third, the sample size (n = 3 biological replicates per condition) is modest, which we aim to increase in future work. In future studies, a more relevant model, a more representative phage-host pair, and phage-tracking techniques will be applied to understand the effects of infection-site morphology on phage mobility and bactericidal ability.

This study identifies substrate texture effects on phage mobility as a determinant of potency, motivating several directions for further study. First, direct characterisation of phage-bacteria interactions in the morphology model will be required. Second, in order to conduct longer-term tests matching clinical phage administration, the model formulation will need to be broadened, including the use of biologically relevant matrices as discussed above. In addition, multiple phage-host pairs and more clinically relevant scenario models will be investigated.

## Conclusion

This study demonstrates that abiotic substrate morphology is a fundamental and previously overlooked factor affecting bacteriophage (phage) bactericidal potency during surface-associated decontamination, with implications for phage therapy where analogous local morphology at the infection site may similarly influence potency. By engineering flat, rough, and porous substrates, a clear hierarchy of bacterial clearance was demonstrated, showing that phage potency was compromised on non-smooth (rough and porous) substrates. Substrate morphology regulates both bacterial spatial distribution and phage mobility, which together affect phage accessibility and the probability of encounter, thereby altering bactericidal performance. These findings highlight the importance of incorporating substrate microarchitecture into phage kinetics and application design, particularly for complex environments such as wound beds and implant-tissue interfaces. Recognising morphology-driven constraints on phage mobility provides a pathway to de-risk phage applications in clinical and industrial settings and may improve the predictive design of dose and dosing interval.
